# Engaging Sexual and Gender Minority Caregivers of Alzheimer’s and Related Dementias: The RISE Project

**DOI:** 10.1002/alz.090137

**Published:** 2025-01-09

**Authors:** Jason D. Flatt, Joel G Anderson, Brittany Klenczar, Cody Yamada, Kari A. Hancock, Krystal R. Kittle, Whitney Wharton

**Affiliations:** ^1^ University of Nevada Las Vegas School of Public Health, Las Vegas, NV USA; ^2^ College of Nursing, University of Tennessee, Knoxville, TN USA; ^3^ University of Massachusetts‐Amherst, Amherst, MA USA; ^4^ Emory University, Atlanta, GA USA

## Abstract

**Background:**

More than 6 million people in the U.S. are currently living with Alzheimer’s disease and related dementias (ADRD), and informal caregivers provide them with more than $270 billion annually in unpaid care. Few studies on ADRD caregiving have engaged caregivers from diverse sexual and gender minorities (SGM), or lesbian, gay, bisexual, transgender, queer, intersex, asexual, and/or another identity.

**Methods:**

This study explores ways to engage and recruit diverse SGM ADRD caregivers into ADRD and aging‐related clinical research. We conducted focus groups and individual interviews with 25 SGM adults who were current, past, or prospective ADRD caregivers. Interviews explored barriers and facilitators to research participation, innovative recruitment strategies, SGM‐specific caregiver needs, and awareness of diverse types of aging and ADRD‐related research. We used thematic analysis to identify key themes.

**Results:**

Caregivers’ ages ranged from 18‐78 years. In terms of race/ethnicity, 48% identified as White, 48% African American, 4% American Indian or Alaska Native, and 10% Hispanic/Latino. For relationships to their care recipients, most reported caring for a parent (47%), followed by a friend (18%), partner/spouse (12%), other relative (12%), or another relationship (11%). The majority provided care for more than a year (52%), followed by 17% providing care for less than a year and 30% expecting to provide care within the next year. We identified five initial themes around recruitment and engagement of SGM ADRD caregivers into research: (1) ensuring inclusion of those from diverse racial/ethnic backgrounds, (2) developing trust through relationship building, (3) safety and privacy measures necessary for SGM identity disclosure, (4) novel recruitment and engagement efforts (media, arts, entertainment), and (5) emphasizing how participating in research supports SGM progress toward equity.

**Conclusions:**

We identified novel ways to recruit and engage diverse SGM ADRD caregivers into research. Future research should explore and test strategies to engage and enhance inclusion of racially/ethnically diverse SGM ADRD caregivers in ADRD research. There is also a need for additional novel strategies to effectively recruit and engage diverse SGM ADRD into research and identify solutions for addressing their health challenges and caregiving needs.

